# A recurrent de novo *ZSWIM6* variant in a Japanese patient with severe neurodevelopmental delay and frequent vomiting

**DOI:** 10.1038/s41439-021-00148-8

**Published:** 2021-05-06

**Authors:** Tomoe Yanagishita, Kaoru Eto, Keiko Yamamoto-Shimojima, Osamu Segawa, Miho Nagata, Yasuki Ishihara, Yohei Miyashita, Yoshihiro Asano, Yasushi Sakata, Satoru Nagata, Toshiyuki Yamamoto

**Affiliations:** 1 Department of Pediatrics, Tokyo Women’s Medical University, Tokyo, 162-8666 Japan; 2 Department of Transfusion Medicine and Cell Processing, Tokyo Women’s Medical University, Tokyo, 162-8666 Japan; 3 Department of Pediatric Surgery, Tokyo Women’s Medical University, Tokyo, 162-8666 Japan; 4 Department of Cardiovascular Medicine, Osaka University Graduate School of Medicine, Suita, 565-0871 Japan; 5 Department of Legal Medicine, Osaka University Graduate School of Medicine, Suita, 565-0871 Japan; 6 Institute of Medical Genetics, Tokyo Women’s Medical University, Tokyo, 162-8666 Japan

**Keywords:** Disease genetics, Neurodevelopmental disorders

## Abstract

A recurrent *ZSWIM6* variant, NM_020928.2:c.2737C>T [p.Arg913*], was identified in a Japanese male patient with severe neurodevelopmental delay, epilepsy, distinctive facial features, microcephaly, growth deficiency, abnormal behavior, and frequent vomiting but without frontonasal or limb malformations. In this patient, distinctive facial features gradually became apparent with age, and severe vomiting caused by gastroesophageal reflux continued even after percutaneous endoscopic gastrostomy.

In 2014, a de novo variant in the zinc finger SWIM-type containing 6 gene (*ZSWIM6*) was commonly identified in four patients with acromelic frontonasal dysostosis (MIM #603671)^1^, a rare disorder characterized by distinct craniofacial, brain, and limb malformations, including frontonasal dysplasia, interhemispheric lipoma, agenesis of the corpus callosum, tibial hemimelia, preaxial polydactyly of the feet, and intellectual disability^2^. In 2017, a different *ZSWIM6* nonsense variant was identified in six patients with severe intellectual disability without frontonasal or limb malformations (MIM #617865)^3^. This evidence indicates that *ZSWIM6* has a clear genotype–phenotype correlation. Recently, we identified the same nonsense variant in a Japanese patient with severe neurodevelopmental delay but without frontonasal or limb malformations.

The male patient is 14 years old. He was born with a birth weight of 2720 g (mean), a length of 47.5 cm (10th–25th centile), and an occipitofrontal circumference (OFC) of 33.0 cm (mean) to healthy parents (a 33-year-old father and a 28-year-old mother). Soon after birth, a heart murmur was detected, and echocardiography showed a small atrial septal defect that spontaneously closed. At 5 months of age, psychomotor developmental delay was noted. At 2 years, he was examined for frequent vomiting, and gastroesophageal reflux (GER) was detected. At that time, he could still not control the movement of his head, and apparent muscular hypotonia was noted. Brain magnetic resonance imaging was performed, but no definite abnormality was identified other than the cavity of the septum pellucidum. At 6 years, he started to show autistic behaviors, including self-injury. He often presented with insomnia. At 12 years, he started to experience generalized seizures. Although there were no paroxysmal discharges on electroencephalography, his epileptic attacks were intractable, despite the combined use of antiepileptic drugs. Owing to frequent vomiting, he underwent percutaneous endoscopic gastrostomy at 12 years; however, he continuously experienced frequent vomiting. Over time, he began to show spasticity in his lower extremities. At present, his height is 130 cm (<3rd centile), his weight is 17.3 cm (<3rd centile), and his OFC is 47.5 cm (<3rd centile), indicating severe growth deficiency and microcephaly. He showed distinctive facial features with right internal strabismus, which were not noted during the infantile period (Fig. 1A). He also showed severe psychomotor developmental delay and had no head control, and he was incapable of meaningful speech. His maintenance of eye contact was poor. Although insomnia was controlled by the prescription of melatonin, his frequent vomiting remained intractable.

**Fig. 1:**
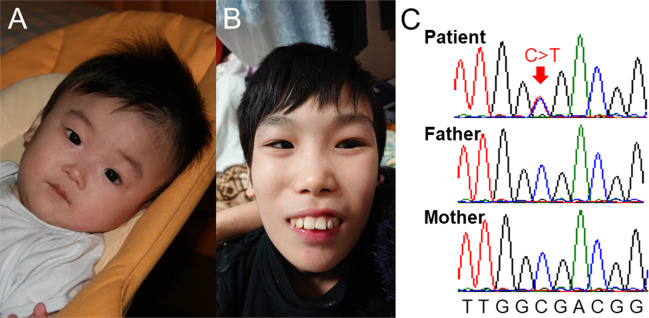
Clinical and laboratory information of the present patient. **A** No distinctive features are noted in infancy. **B** Distinctive features, including a prominent forehead, prominent supraorbital ridges, and prominent cheeks, are shown at 14 years. These photos were provided by his family with written informed consent. **C** Results of Sanger sequencing. The patient shows a de novo variant (C>T).

Prior conventional chromosomal G-banding showed a normal male karyotype of 46XY, and subsequently performed chromosomal microarray testing performed as described previously showed no abnormal findings^4^. Thus, this patient was enrolled in the research project “Initiative on Rare and Undiagnosed Diseases (IRUD)”^5,6^. This study was performed in accordance with the Declaration of Helsinki and was approved by the ethics committee of our institution. After obtaining written informed consent, we collected blood samples from the patient and both parents. Genomic DNA was extracted from the peripheral blood of the individuals using a standard protocol. Exome sequencing was performed using trio samples, including parental samples, as described previously^7^. Finally, we identified a heterozygous variant, NM_020928.2(ZSWIM6):c.2737C>T [p.Arg913*], the same variant reported by Palmer et al.^3^. Because neither parent showed this variant, it was considered de novo. Standard PCR-Sanger sequencing confirmed the de novo occurrence of this variant (Fig. 1B). No other variants that might be related to the clinical features of this patient could be identified. The clinical features of the present patient are summarized in Table 1, together with the clinical data provided by Palmer et al.^3^. As shown, the patient showed clinical features that were similar to those of the other patients. Therefore, we determined that the identified *ZSWIM6* variant was responsible for his clinical features.

**Table 1:** Clinical features of the present patient in comparison with previously reported patients.

	Present patient	Palmer et al. ^3^
Parental ages at birth of child (years)	Paternal 33/Maternal 28	25–34
Evidence of mosaicism	No (allelic depth 8/17 [47%]^a^)	No
Age (years)	14	3–29
Gender	Male	3/7 male (43%)
Level of intellectual disability	Severe	7/7 severe profound
Occipitofrontal circumference	Progressive microcephaly	3/7 progressive microcephaly
Infantile hypotonia/delayed	+	7/7 (100%)
Autism spectrum disorder	+	5/7 (71%)
Communication	−	6/7 non-verbal or only few words (86%)
Ambulation	−	5/7 ambulant (71%) with wide-based gait
Temperament/behavior	Self-injury	4/7 hyperactivity (57%)
Epilepsy	+	4/7 seizure/ possible seizure disorder (57%)
Progressive spasticity	+	3/7 (43%)
Movement disorder	Head tics	6/7 (86%)
Ophthalmological features	Strabismus	5/7 (71%)
Brain magnetic resonance imaging	Cavity of septum pellucidum	1/7 cortical atrophy (14%)
Additional neurological features	Truncal hypotonia	5/7 (71%)
Gastro-intestinal symptoms	Frequent vomitting	6/7 (86%) significant symptoms
Distinctive facial features	+	7/7 (100%)

This table is referred to the table provided in the report by Palmer et al.^3^.

^a^ Allelic depth means depth of a rare variant in total reads at the indicated position.

*ZSWIM6* is located on chromosome 5q12.1 and consists of 14 exons. Using mice, it was revealed that *Zswim6* is initially expressed widely during embryonic brain development, suggesting a critical role of this gene in neuronal development^8^. There are some reports of chromosomal microdeletions in the *ZSWIM6* region^9,10^; however, patients with microdeletions involving *ZSWIM6* show milder neurodevelopmental disorders than patients with the p.Arg913* *ZSWIM6* variant. This indicates that the haploinsufficiency of *ZSWIM6* is not a mechanism. The p.Arg913* *ZSWIM6* variant lies within the penultimate exon 13, 48 bp upstream of the last exon/exon junction, suggesting that the *ZSWIM6* mRNA encoding this pretermination codon may escape nonsense-mediated decay. Thus, it is considered that the p.Arg913* *ZSWIM6* variant may result in a dominant-negative effect due to the production of a truncated ZSWIM6 protein that lacks the Sin-3-like domain in exon 14.

The present patient showed no distinctive facial features during infancy; however, his facial appearance changed with age, and the characteristics gradually appeared, as shown in Fig. 1A. This finding is also common with patients reported by Palmer et al.^3^. The most prominent symptom in the present patient was frequent vomiting. Palmer et al. reported that 4/7 patients (57%) showed GER, and two of them received gastrostomy^3^. Similarly, the present patient also underwent percutaneous endoscopic gastrostomy; however, severe vomiting persisted. Therefore, we are currently considering the placement of an elemental diet tube into the duodenum to avoid vomiting. It is unknown why patients with the p.Arg913* *ZSWIM6* variant show such severe GER. The mechanism underlying severe GER and an appropriate treatment approach should be identified in the future.

## Data Availability

The relevant data from this Data Report are hosted at the Human Genome Variation Database at 10.6084/m9.figshare.hgv.2999.
